# Analysis of three pigment epithelium-derived factor gene polymorphisms in patients with exudative age-related macular degeneration

**Published:** 2009-02-16

**Authors:** Dietmar Mattes, Anton Haas, Wilfried Renner, Iris Steinbrugger, Yosuf El-Shabrawi, Andreas Wedrich, Christoph Werner, Otto Schmut, Martin Weger

**Affiliations:** 1 Department of Ophthalmology, Medical University of Graz, Austria; 2 Clinical Institute of Medical and Chemical Laboratory Diagnostics, Medical University of Graz, Austria

## Abstract

**Purpose:**

Exudative age-related macular degeneration (exudative AMD) is a common vision-threatening disease, with both environmental and genetic factors contributing to its development. Recently, homozygosity for the 72Met variant of the pigment epithelium-derived factor (*PEDF*) Met72Thr gene polymorphism (rs1136287) was identified as a novel risk factor for exudative AMD in Chinese patients from Taiwan. The role of this polymorphism, however, has not yet been determined in a white European population. In addition, two other *PEDF* gene polymorphisms, −5736T>C (rs12150053) and −5304C>T (rs12948385), have been associated with increased risk of diabetic retinopathy, but have not yet been studied among patients with exudative AMD. The purpose of the present study was thus to investigate a hypothesized association between these *PEDF* polymorphisms and the presence of exudative AMD in a white European population.

**Methods:**

The present case-control study comprised 269 patients with exudative AMD and 155 control subjects. Genotypes of the *PEDF* polymorphisms were determined by 5′-exonuclease assays (TaqMan).

**Results:**

*PEDF* genotype and allele frequencies were not significantly different between AMD patients and control subjects. The two promoter polymorphisms, −5736T>C (rs12150053) and −5304C>T (rs12948385), were in complete association. Presence of the homozygous *PEDF* 72 Met/Met genotype was associated with a nonsignificant odds ratio of 1.00 (95% confidence interval: 0.67–1.49, p=0.99). Similarly, presence of the homozygous *PEDF* −5736 TT genotype or −5304 CC genotype was associated with a nonsignificant odds ratio of 0.99 (95% confidence interval: 0.56 - 1.75, p=0.97). Both promoter polymorphisms were in linkage disequilibrium with the Met72Thr (rs1136287) polymorphism (D'=0.83) and formed three common and one rare haplotype. Haplotype frequencies were similar between AMD patients and control subjects (p>0.05).

**Conclusions:**

Our data suggest that none of the investigated *PEDF* polymorphisms is likely a major risk factor for exudative AMD in a white European population.

## Introduction

Exudative age-related macular degeneration (exudative AMD) is a major cause of severe visual impairment in patients older than 50 years [1–3]. An impaired balance between pro- and antiangiogenic factors has previously been implicated in the development of choroidal neovascularization in AMD [4–6].

Pigment epithelium-derived factor (PEDF), a 50 kDa glycoprotein belonging to the serine proteinase inhibitor family [7–10], is a potent antiangiogenic factor [11,12] and exerts neurotrophic and neuroprotective effects [8,13]. It is synthesized by several different cell types including retinal pigment epithelium (RPE) cells and photoreceptors [14]. Several lines of evidence indicate a role of PEDF in the pathogenesis of exudative AMD. First, immunohistochemical studies have revealed significantly reduced immunoreactivity for PEDF in both RPE cells and in Bruch’s membrane of AMD eyes compared with healthy control eyes [6,15]. Second, vitreous PEDF concentrations were found to be significantly decreased in eyes with exudative AMD [16]. Additional evidence comes from an animal laser injury model showing an inverse correlation between PEDF expression and formation of choroidal neovascularizations [17,18]. Finally, the administration of recombinant natural PEDF or adenoviral vector-delivered PEDF has been found either to inhibit the development of choroidal neovascularizations or to reduce its extent [19–21].

In 2005, Yamagishi et al. [22] proposed the hypothesis that a *PEDF* gene polymorphism, which is characterized by a methionine to threonine substitution at amino acid position 72 of *PEDF* (PEDF Met72Thr [PEDF 311T>C], rs1136287) [23], might be a genetic marker for AMD. Indeed, Lin et al. [24] only recently identified this polymorphism as a novel risk factor for exudative AMD in a Taiwan Chinese population. So far, this finding has not yet been replicated in a white European population. This, however, is essential to draw firm conclusions on the potential contribution of gene polymorphisms to exudative AMD risk in populations of different ethic origin. Two other *PEDF* polymorphisms, −5736T>C (rs12150053) and −5304C>T (rs12948385), have only recently been associated with diabetic retinopathy, but have not yet been studied in AMD patients [25]. The purpose of the present study was thus to investigate a hypothesized association between the aforementioned *PEDF* polymorphisms and exudative AMD in a white European population.

## Methods

The study comprised 269 patients with exudative AMD and 155 control participants who were of European origin and living in the same geographical area in the southern part of Austria. All participants were seen at the local Department of Ophthalmology, Medical University of Graz, and gave written informed consent before enrollment. The study was conducted according to the Austrian Gene Technology Act and the guidelines of the local Ethics Committee.

Exudative AMD was diagnosed by ophthalmoscopic fundus examination, followed by fluorescein/indocyanine angiography revealing choroidal neovascularizations. Exclusion criteria comprised the presence of choroidal polypoidal vasculopathy or secondary choroidal neovascularizations due to pathologic myopia (>6 diopters, spherical equivalent), inflammatory or infectious chrorioretinal diseases, trauma, angioid streaks or hereditary diseases.

Each control participant underwent a detailed eye examination that included fundus examination. Exclusion criteria were defined as any evidence of age-related maculopathy (drusen as well as pigmentary changes), macular hemorrhages of any cause, or media opacities leading to impaired visualization of the macula.

### Genotype determination

Venous blood drawn from the antecubital vein was collected in ethylene diamine tetraacetic acid tubes. Genomic DNA was isolated from whole blood using a commercial kit (QIA_AMP DNA blood mini kit; Qiagen, Vienna, Austria) and stored at −20 °C. *PEDF* genotype were determined by 5′-exonuclease assays (TaqMan). Primers and probes are summarized in Table 1. Fluorescence was measured in a lambda Fluoro 320 Plus plate reader (MWG Biotech AG, Penzberg, Germany) using excitation/emission filters of 485/530 nm for probes labeled with fluorescent dye FAM and 530/572 nm for probes labeled with fluorescent dye VIC. The data were exported into Excel format, depicted and analyzed as a scatter plot. The technicians responsible for genotyping were blinded for case/control status.

**Table 1:** Sequences of primers and probes used for the determination of the *PEDF* genotypes.

**PEDF polymorphism**	**Primers (5′-3′) and probes**
−5736C>T (rs12150053)	F: CAGCCTGGGTGACAGAGT
	R: AACCTTAGGTCAATGTATCACACTGTTC
	WT-probe: VIC-TTTCCAGTGGAGACTC-NFQ
	mutant probe: FAM-TTTTCCAATGGAGACTC-NFQ
−5304C>T (rs12948385)	F: CAACACACCTGGGTAATTTTGTTTGT
	R: ACCTGAGGTCAGGAGTTCGA
	WT-probe: VIC-TTCACCGTGTTGGCTAG-NFQ
	mutant probe: FAM-TTCACCGTGTTGACTAG-NFQ
Met72Thr (rs1136287)	F: CCAACTTCGGCTATGACCTGTAC
	R: GAGACAGGAGCACGTTGGT
	WT-probe: VIC-CCAGCATGAGCCCCA-NFQ
	mutant probe: FAM-CAGCACGAGCCCCA-NFQ

PEDF genotypes were determined by 5′-exonuclease assays (TaqMan) using probes labeled with fluorescent dyes VIC or FAM. Abbreviations used in the table: F represents forward primer, R represents reverse primer, WT represents wild-type.

### Statistics

SPSS for Windows (release 14.0; SPSS, Inc.) was used for statistical analyses. Continuous variables were analyzed by *t*-test and presented as mean±standard deviation (SD). Categorical variables are presented as percentages and were compared as chi-square test. Odds ratios (OR) and 95% confidence intervals (CI) were determined by logistic regression analysis. The criterion for statistical significance was p<0.05.

## Results

Clinical characteristics of AMD patients and control participants are presented in Table 2. Table 3 shows *PEDF* genotype distribution in AMD patients and controls. In both groups, the observed genotype distributions were in line with those predicted by the Hardy–Weinberg equilibrium. *PEDF* genotype and allele frequencies were not significantly different between AMD patients and controls (Table 3). The two promoter polymorphisms, −5736T>C (rs12150053) and −5304C>T (rs12948385), were in complete association.

**Table 2:** Clinical characteristics of exudative AMD patients and controls.

**Clinical characteristics**	**Exudative AMD patients (n=269)**	**Controls (n=155)**
Females	178 (66.2)	86 (55.5)
Mean age±SD (years)	78.4±7.0	77.4±6.5
Range (years)	55.8–94.1	53.2–91.1
Body mass index *	26.3±3.9	26.7±4.0
Arterial hypertension	161 (59.9)	111 (71.6)
Hypercholesterolemia	193 (71.7)	101 (65.2)
Diabetes mellitus	26 (9.7)	22 (14.2)
Myocardial infarction	19 (7.1)	13 (8.4)
History of smoking	75 (27.9)	34 (21.9)

Numbers are given as n (%). Abbreviations: age-related macular degeneration (AMD); standard deviation (SD). The asterisk indicates that the data on body mass index were available from 205 AMD patients and 151 control subjects.

**Table 3:** *PEDF* genotypes in exudative AMD patients and controls.

**Polymorphism**	**Genotype**	**Exudative AMD patients (n=269)**	**Controls (n=155)**	**p**
Met72Thr (rs1136287)	Met/Met	111 (41.3%)	64 (41.3%)	0.81
	Met/Thr	123 (45.7%)	74 (47.7%)	
	Thr/Thr	35 (13.0%)	17 (11.0%)	
	MAF	0.359	0.348	0.76
−5736T>C (rs12150053)	TT	108 (40.1%)	67 (43.2%)	0.71
	TC	117 (43.5%)	61 (39.4%)	
	CC	44 (16.4%)	27 (17.4%)	
	MAF	0.381	0.371	0.77
−5304C>T (rs12948385)	CC	108 (40.1%)	67 (43.2%)	0.71
	CT	117 (43.5%)	61 (39.4%)	
	TT	44 (16.4%)	27 (17.4%)	
	MAF	0.381	0.371	0.77

Data indicate the number of eyes with and without exudative age-related macular degeneration (AMD), respectively. Numbers are given as n (%). Neither PEDF genotypes nor minor allele frequencies (MAF) were significantly associated with exudative AMD.

Presence of the homozygous *PEDF* 72 Met/Met genotype was associated with a nonsignificant OR of 1.00 (95% CI: 0.67–1.49, p=0.99). Similarly, presence of the homozygous PEDF−5736 TT genotype or −5304 CC genotype was associated with a nonsignificant OR of 0.99 (95% CI: 0.56–1.75, p=0.97). The observed OR was not substantially altered after adjustment of smoking habits (data not shown).

Both promoter polymorphisms were in linkage disequilibrium with the Met72Thr (rs1136287) polymorphism (D'=0.83). In total, three common haplotypes and one rare haplotype were formed by the *PEDF* polymorphisms investigated in the present study. Haplotype frequencies were similar between AMD patients and controls (Table 4).

**Table 4:** *PEDF* haplotype frequencies in exudative AMD patients and controls.

**Haplotypes formed by**	**Exudative AMD patients(n=269)**	**Controls (n=155)**	**p**
C-T-Thr	0.361	0.338	0.51
T-C-Met	0.338	0.316	0.5
T-C-Thr	0.28	0.294	0.31
C-T-Met	0.02	0.033	0.26

Frequencies of haplotypes formed by PEDF polymorphisms −5736T>C (rs12150053), −5304C>T (rs12948385) and Met72Thr (rs1136287). Frequencies of haplotypes formed by PEDF polymorphisms −5736T>C (rs12150053), −5304C>T (rs12948385) and Met72Thr (rs1136287) did not significantly differ between patients and controls. Frequencies and p values were calculated using the HaploView 4.0 software.

## Discussion

Exudative AMD has previously been shown to have a strong genetic component [26–39]. Only recently, a significantly increased prevalence of the *PEDF* 72 Met/Met genotype was reported in a Taiwan Chinese population, yielding an OR of 3.9 for exudative AMD [24]. This, however, has not yet been confirmed in other populations of different ethnic origin.

To the best of our knowledge, the present study is the first to investigate the potential role of this polymorphism in a white European population. In contrast to the findings of Lin et al. [24], homozygosity for the *PEDF* 72Met allele was not found to be significantly more prevalent in AMD patients compared with controls. Importantly, our study had a statistical power of 0.8 to detect or exclude an OR greater than or equal to 1.8. Thus, our data strongly suggest that the *PEDF* Met72Thr polymorphism itself is unlikely a major risk factor for exudative AMD in a white European population.

Interestingly, the *PEDF* 72Met allele frequency was 0.652 in our control group of white European subjects, which is substantially higher than the *PEDF* 72Met allele frequency of 0.311 found in a Taiwan Chinese population [24]. This finding indicates that genotype distributions of this polymorphism vary widely between different populations and thus underlines the importance of performing genetic association studies in various ethnicities.

As *PEDF* expression in RPE cells is also influenced by other factors such as oxidative stress [40], our finding that the *PEDF* Met72Thr polymorphism is not associated with exudative AMD risk in an European population does not argue against a role of *PEDF* in AMD. Beside the Met72Thr polymorphisms, other *PEDF* gene variations may be associated with exudative AMD. Recently, two polymorphisms in the *PEDF* promoter, −5736T>C (rs12150053) and −5304C>T (rs12948385), but not the Met72Thr polymorphism, were found to be associated with diabetic retinopathy [25,41]. The present study is also the first to investigate the potential association of these polymorphisms with exudative AMD. A major finding of our study was that the two polymorphisms did not confer a significantly increased risk for exudative AMD in a white European population.

Currently, the functionality of the *PEDF* polymorphisms investigated in the present study is unclear. A variety of putative binding sites for transcription factors and two Alu repetitive sequences have been described in the *PEDF* promoter [9], but the two promoter polymorphisms (−5736T>C [rs12150053] and −5304C>T [rs12948385]) we investigated do not affect any of these putative binding sites. The Met72Thr polymorphism lies at the end of a helix domain of the PEDF protein and results in the exchange of a hydrophobic amino acid (methionine) by a polar amino acid (threonine) [23]. Nevertheless, the functional consequences of this exchange are currently unknown. Further studies investigating the influence of these polymorphisms on the expression and function of the PEDF protein will be necessary to clarify their functionality.

Previous studies have clearly demonstrated that PEDF itself is able to inhibit the development of choroidal neovascularizations [19–21]. Considering the antiangiogenic and anti-vasopermeability effects of PEDF [11,12], it remains to be determined whether gene polymorphisms affecting the expression of *PEDF* in the chorioretinal tissue may modulate the efficacy as well as frequency of anti-vascular endothelial growth factor treatment. Thus, further studies focusing on the identification of functional *PEDF* polymorphisms and their potential association with treatment outcome might be of great interest.
